# Preparation and mechanism research of Ni-Co supported catalyst on hydrogen production from coal pyrolysis

**DOI:** 10.1038/s41598-019-44271-7

**Published:** 2019-07-08

**Authors:** Lei Zhang, Xin Wen, Tingting Kong, Lei Zhang, Long Gao, Lintian Miao, Yong-hui Li

**Affiliations:** 1 School of Geology and Environment, Xi’an University of Science and Technology, Xi’an, 710054 China; 2 Xi’an Shiyou University, Xi’an, 710065 China; 3 China National Heavy Machinery Research Institute Co, Ltd., Xi’an, 710032 China; 4 China University of Mining & Technology, Beijing, 100083 China; 5 Key Laboratory of Coal Resources Exploration and Comprehensive Utilization, Ministry of Land and Resources, 710021 Xi’an, China

**Keywords:** Atmospheric chemistry, Pollution remediation

## Abstract

The combustible gas produced by coal pyrolysis can be used as a clean fuel to reduce environmental pollution caused by coal combustion. In order to improve the efficiency of coal pyrolysis to prepare flammable gases, Ni-Co supported pyrolysis catalysts were prepared by equal volume impregnation method using the pyrolysis coke of different pyrolysis final temperatures as the carrier in this study. The effects of different pyrolysis final temperatures and different metal loadings on pyrolysis products were studied. The cracking mechanism of the catalysts was characterized by BET, XRD, XPS and SEM. The results show that: (1) the catalytic efficiency of pyrolysis coke catalyst is mainly related to the metal type and loading of the additive. (2) The optimum preparation conditions for the supported catalyst are: use pyrolysis coke with 750 °C final pyrolysis temperature as the carrier, the loading amount of Ni is 5%, and the loading amount of Co is 8%.

## Introduction

Coal energy has made an indispensable contribution to the development of human society; however, we have also paid a heavy price for this at the same time. The direct combustion of coal is the chief culprit of environmental pollution. According to the statistics, 90% of SO_2_, 67% of NO_X_ and 82% of acid rain and other soot pollutants in the atmosphere are produced by direct combustion of coal^1^. These pollutants not only destroy the ecological balance, but also cause great harm to the human body, which seriously restricts the sustainable development of the world economy. Therefore, human beings must seek a new clean, safe and reliable sustainable energy system. In the many energy fields, pure hydrogen becomes the focus of research because it not only has a high heat value, but also does not pollute the environment as it only produces water after combustion^2^.

Coal cracking generates radicals, and then some of the radicals polymerize to form semi-coke, the remaining part form primary volatiles when it is stabilized; primary volatiles undergo secondary cracking and polymerization during diffusion into the gas phase.

The organic molecules in coal are connected to each other by bridge, a large number of weak bonds are interrupted to form free radicals with the temperature increasing. A part of the side chains with stronger chemical bond also starts to cleave when the temperature rises to a certain value, which leads the concentration of free radicals increasing. In this process, the small molecular compound and radicals enter the gas phase from the coal plastid by diffusion, and a secondary reaction occurs while moving in the diffusion process, finally combined with each other to form heavy tar and semi-coke due to the polycondensation^3–6^.

Coal gasification refers to the process of carbon oxide, hydrogen and methane produced from coal or semi-coke with catalyst by heterogeneous reaction, it is mainly the interaction between carbon in a solid fuel with oxygen, water vapor, carbon dioxide, and hydrogen in the gas phase. It can also be said that the gasification process of coal is a process of converting unusable solids in coal into industrial fuel, city gas and chemical raw material gas^7–10^.

Coal gasification process can be divided into two types: homogeneous and heterogeneous reactions. That is, a heterogeneous gas-solid phase reaction and a homogeneous gas-gas phase reaction. The composition of the generated gas depends on the integrated process of these reactions. The main reactions are as follows:

First-order reactions:

1 $$\begin{array}{cc}{\rm{C}}+{{\rm{O}}}_{2}\to {{\rm{CO}}}_{2}+{\rm{\Delta }}{\rm{H}} & \,{\rm{\Delta }}{\rm{H}}=-\,394.1\,\text{kJ}/\text{mol}\end{array}$$

2 $$\begin{array}{cc}{\rm{C}}+{{\rm{H}}}_{2}{\rm{O}}\to {\rm{CO}}+{{\rm{H}}}_{2}+{\rm{\Delta }}{\rm{H}} & \,{\rm{\Delta }}{\rm{H}}=135.0\,\text{kJ}/\text{mol}\end{array}$$

3 $$\begin{array}{cc}{\rm{C}}+1/2{{\rm{O}}}_{2}\to {\rm{CO}}+{\rm{\Delta }}{\rm{H}} & \,{\rm{\Delta }}{\rm{H}}=-\,110.4\,\text{kJ}/\text{mol}\end{array}$$

4 $$\begin{array}{cc}{\rm{C}}+2{{\rm{H}}}_{2}{\rm{O}}\to {{\rm{CO}}}_{2}+2{{\rm{H}}}_{2}+{\rm{\Delta }}{\rm{H}} & \,{\rm{\Delta }}{\rm{H}}=96.6\,\text{kJ}/\text{mol}\end{array}$$

5 $$\begin{array}{cc}{\rm{C}}+2{{\rm{H}}}_{2}\to {{\rm{CH}}}_{4}+{\rm{\Delta }}{\rm{H}} & \,{\rm{\Delta }}{\rm{H}}=-\,84.3\,\text{kJ}/\text{mol}\end{array}$$

$$\begin{array}{cc}{{\rm{H}}}_{2}+1/2{{\rm{O}}}_{2}\to {{\rm{H}}}_{2}{\rm{O}}+{\rm{\Delta }}{\rm{H}} & \,{\rm{\Delta }}{\rm{H}}=-\,245.3\,\text{kJ}/\text{mol}\end{array}$$

Second-order reactions:

6 $$\begin{array}{cc}{\rm{C}}+{{\rm{CO}}}_{2}\to 2{\rm{CO}}+{\rm{\Delta }}{\rm{H}} & \,{\rm{\Delta }}{\rm{H}}=173.3\,\text{kJ}/\text{mol}\end{array}$$

7 $$\begin{array}{cc}{\rm{2CO}}+{{\rm{O}}}_{2}\to 2{{\rm{CO}}}_{2}+{\rm{\Delta }}{\rm{H}} & \,{\rm{\Delta }}{\rm{H}}=-\,566.6\,\text{kJ}/\text{mol}\end{array}$$

8 $$\begin{array}{cc}{\rm{CO}}+{{\rm{H}}}_{2}{\rm{O}}\to {{\rm{H}}}_{2}+{{\rm{CO}}}_{2}+{\rm{\Delta }}{\rm{H}} & \,{\rm{\Delta }}{\rm{H}}=-\,38.4\,\text{kJ}/\text{mol}\end{array}$$

9 $$\begin{array}{cc}{\rm{CO}}+3{{\rm{H}}}_{2}\to {{\rm{CH}}}_{4}+{{\rm{H}}}_{2}{\rm{O}}+{\rm{\Delta }}{\rm{H}} & \,{\rm{\Delta }}{\rm{H}}=-\,219.3\,\text{kJ}/\text{mol}\end{array}$$

10 $$\begin{array}{cc}3{\rm{C}}+2{{\rm{H}}}_{2}{\rm{O}}\to {{\rm{CH}}}_{4}+2{\rm{CO}}+{\rm{\Delta }}{\rm{H}} & \,{\rm{\Delta }}{\rm{H}}=185.6\,\text{kJ}/\text{mol}\end{array}$$

11 $$\begin{array}{cc}3{\rm{C}}+2{{\rm{H}}}_{2}{\rm{O}}\to {{\rm{CH}}}_{4}+{{\rm{CO}}}_{2}+{\rm{\Delta }}{\rm{H}} & \,{\rm{\Delta }}{\rm{H}}=12.2\,\text{kJ}/\text{mol}\end{array}$$

According to the products of the above reactions, the coal gasification process can be expressed by the following formula:

$${\rm{Coal}}\,\underset{{\rm{pressure}},\,{\rm{catalyst}}}{\overset{{\rm{High}}\,{\rm{temperature}}\,{\rm{and}}}{\longrightarrow }}\,{\rm{C}}+{{\rm{CH}}}_{4}+{\rm{CO}}+{{\rm{CO}}}_{2}+{{\rm{H}}}_{2}+{{\rm{H}}}_{2}{\rm{O}}$$

So if the coal is used as a source of hydrogen rather than directly burned, we could convert the coal into a clean and highly efficient gas fuel, at the same time, centralized treatment and conversion of harmful substances in coal can be realized, which can avoid the environmental pollution and high treatment cost caused by the decentralized combustion of coal^11–13^. In this way, not only the utilization efficiency of coal is improved, but also the contradiction between coal combustion and environmental problems is solved, and the pace of universal utilization of hydrogen energy is also accelerated^14^.

In this paper, pyrolysis coke is used as a catalyst carrier to prepare Ni-Co supported catalyst to improve the efficiency of preparing combustible gas by coal pyrolysis, creating economic value while solving environmental problems.

## Experiments

### Experimental materials and methods

This experiment uses Yimin lignite as raw material.

Screen Yimin lignite with a particle size of 3 to 5 mm and put it into a sealed bag for use. Industrial analysis and elemental analysis of Yimin lignite are shown in Table 1.

**Table 1:** Industrial analysis and elemental analysis of Yimin lignite.

	Water	Ash	Volatiles	C	H	N
The proportion (%)	15.23	16.58	36.56	58.93	4.093	1.136

The equipment for elemental analysis of coal samples is: the Organic element was detected and analyzed by the Vario EL III Element Analyzer (Produced by Elementar, Germany).

The equipment for industrial analysis: water: Weigh a coal sample with a particle size of 0.2 MM, Place it in a dry box pre-passed with dry nitrogen and heated to (105~110) °C for 2 h, take out it and placed in a desiccator, cooled to room temperature (20 min), and weighed.

Ash: put the coal sample into the muffle furnace, heat it to (815 ± 10) °C at a certain speed, ashing and burn until the mass is constant. The ash is the ratio of the residue quality to the coal sample quality.

Volatile matter: place the coal into a porcelain crucible with a lid. At (900 ± 10) °C, Isolated air, heating for 7 min, the volatile matter of the coal sample is:

$$\frac{{m}_{totallose}-{m}_{water}}{{m}_{coal}}$$

Before distillation, the mass of the dry distillation tube was 97.411 g; the mass of the conical flask was 125.630 g, the mass of the coal sample was 20.000 g, and the total mass of the dry distillation tube and the coal sample was 117.409 g.

After distillation, the total mass of the dry distillation tube and the semi-coke was 110.097 g, the total mass of the conical flask, tar and water was 129.538 g; the cumulative gas flow rate was 2.3 L, and the volume of distilled water was 3 mL. The mass of the volatile gas was 7.312 g (117.409 g–110.097 g), and the tar and water quality were 4.178 g (129.538 g–125.360 g). The gas mass was 2.9739 g (2.3 L × 1.293 g/L, assuming gas density was air density, ρ = 1.293 g/l, and gas mass was calculated according to the formula m = ρ × V). The total mass of tar, water and gas was 7.1519 g (4.178 g + 2.9739 g), so the acceptable deviation of coal sample quality reduction was only 0.1601 g (7.312 g–7.1519 g), ie 0.8% (0.1601 g/20 g). In this study, the volatile content of Yimin lignite was 36.56% (7.312 g/20 g), and the water content was 15.23% (3.046 g/20 g).

### Preparation of the pyrolysis coke catalyst

#### Preparation of the pyrolysis coke with different final pyrolysis temperature

A certain mass of coal sample was weighed and placed in a pyrolysis furnace, and N_2_ was introduced at a gas flow rate of 40 ml/min. The pyrolysis furnace was separately heated to 450 °C, 550 °C, 650 °C and 750 °C, and the coal sample was heated until no gas was generated. Then cool the coke to room temperature in a nitrogen atmosphere to obtain a pyrolysis coke having a final pyrolysis temperature of 450 °C, 550 °C, 650 °C, and 750 °C, respectively.

#### Preparation of single metal supported catalyst

The impregnation solution was prepared by using nickel nitrate with a purity of 98% and pyrolysis coke catalyst with a loading of 5%, 8% and 10% was prepared by equal volume impregnation method. The mass of nickel nitrate required is calculated according to the amount of pyrolysis coke and melted by heating. Then weigh and mix the coke with the melted nickel nitrate evenly, and then dry in an oven after standing for 24 hours. Determine the optimal loading of the nickel nitrate by experiment after obtaining the single metal supported catalyst.

#### Preparation of bimetallic supported catalyst

The immersion liquid was prepared by using cobalt nitrate with a purity of ≥99%. The single metal catalyst with the optimum nickel nitrate loading was used as the primary catalyst, and the bimetallic supported catalyst with cobalt nitrate loading of 3%, 5% and 8% was prepared by the equal volume impregnation method. Then it is treated with optimal plasma modification-baking conditions and the optimum bimetallic loading is determined experimentally.

#### The evaluation device for the catalyst

The evaluation device for the catalyst in this study is mainly: Determination of the composition of the pyrolysis gas by gas chromatography (GC1100), the Organic element in the coal was detected and analyzed by the Vario EL III Element Analyzer (Produced by Elementar, Germany). The XRD pattern was obtained by XD-3 X-ray diffraction analyzer (manufactured by Beijing General Instrument Co., Ltd.), X-ray photoelectron analysis was used to analyze the structure and composition on the pyrolysis surface. The morphology of the pyrolysis coke catalyst was amplified by scanning electron microscopy (SEM) and observed. Its model is SM-6460LV (produced by Japan Electron Beam Co., Ltd.).

### The experimental device

**Figure 1:**
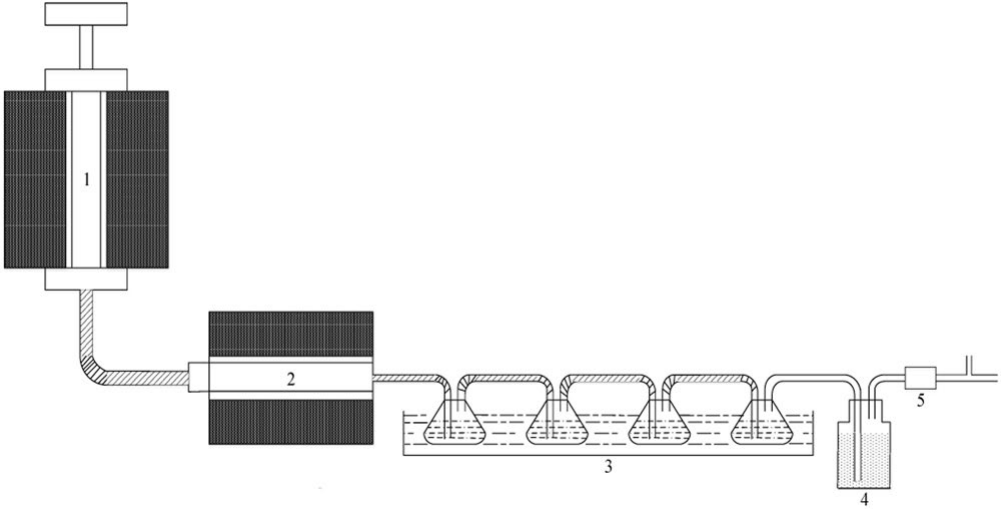
Process flow in this experiment. (1) The first-stage pyrolysis furnace. (2) The second-stage pyrolysis furnace. (3) The acetone washing gas bottle. (4) The dry silica gel. (5) The mass flow meter.

The process flow used in this experiment to determine the activity of the catalyst is shown in Fig. 1.

In the experiment, weigh 20 g coal sample and place it in first-stage furnace, weigh 4 g catalyst and place it into second-stage furnace. Raise the temperature of the second stage furnace to 400 °C, and the temperature of first stage furnace to 500 °C. The tar and gas were produced by the coal pyrolysis in first-stage furnace and the tar is cracked by the pyrolysis coke catalyst in the second-stage furnace. The mixed gas flow enters the tar collecting device to ensure the tar in gas is completely collected by the acetone in the device, and the remaining mixed gas is dried by the gas drying bottle and the gas volume is recorded by the flow meter. The mixture of acetone and tar in tar collecting device is separated by rotary evaporator, and the mass of the tar is accurately weighed. The catalytic effect of the catalyst was determined by comparing the gas production between the blank groups experiment^15^. In a word, the pyrolysis coke in the second stage furnace acts as a catalyst to crack the heavy tar produced by the coal sample which is in the first stage furnace, and the catalyst will cause the heavy tar to be cracked into more light tar and hydrogen-rich gas.

(In this experiment, we collect the tar with a conical flask containing acetone, collect the gas with a gas bag, and separate the tar in the acetone with a rotary evaporator when the experiment completed. Then, weighed the mass of the tar, determine the proportion of H_2_, CO, and CH_4_ in the gas by the gas chromatography, and the volumes of H_2_, CO, and CH4 are calculated).

## Discussion and Results

### Effect of different final temperature on pyrolysis products

**Figure 2:**
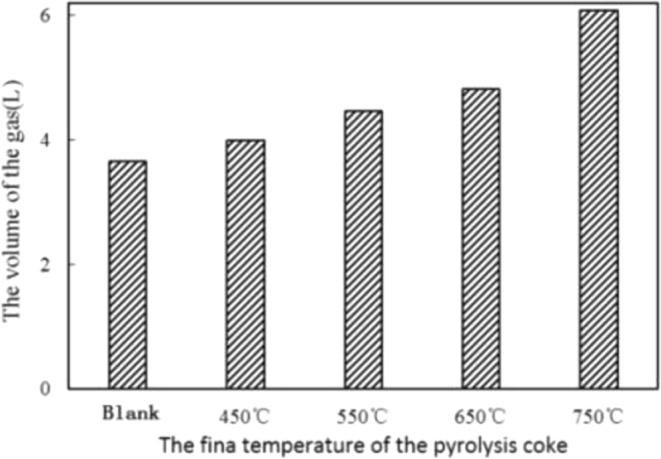
Effect of final temperature on gas yield by tar cracking.

To study the effect of pyrolysis coke catalyst on pyrolysis products under different pyrolysis final temperature, put 20 g coal sample in first-stage furnace, set the temperature to 500 °C, and put 4 g pyrolysis coke catalyst with different final temperature in the second-stage furnace. Compare the gas yield in the obtained products, and choose the best pyrolysis coke catalyst for the subsequent experiments. The experimental results are shown in Fig. 2. It can be known that the gas yield of tar cracking is the highest when the pyrolysis coke catalyst prepared at the final pyrolysis temperature of 750 °C, so the pyrolysis coke catalyst with the final temperature of 750 °C is selected for subsequent experiments.

**Figure 3:**
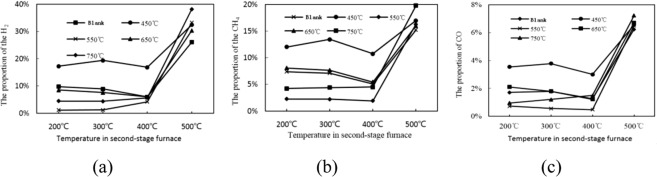
Effect of final pyrolysis temperature on the yield of H_2_, CH_4_ and CO.

Combined with Fig. 3, we can know that when the temperature is 200~400 °C, the gas yield by tar cracking is not significantly improved. When the temperature reaches 500 °C, the gas yield is greatly increased, and the proportion of H_2_, CH_4_ and CO has also increased significantly. When the pyrolysis coke catalyst is placed in the second-stage furnace, the gas yield by tar cracking is increased. Compared with the blank group, the gas yield is increased by 52.6%, and the proportion of the flammable gas (H_2_, CH_4_ and CO) is also increased. This is because the activity of the pyrolysis coke catalyst increases with the temperature increasing, and a part of the heavy tar adsorbed on its surface is cleaved into a light component and gas^16^, so that the gas yield increases, and the tar yield decreases.

### Activity evaluation of single metal supported pyrolysis coke catalyst

**Figure 4:**
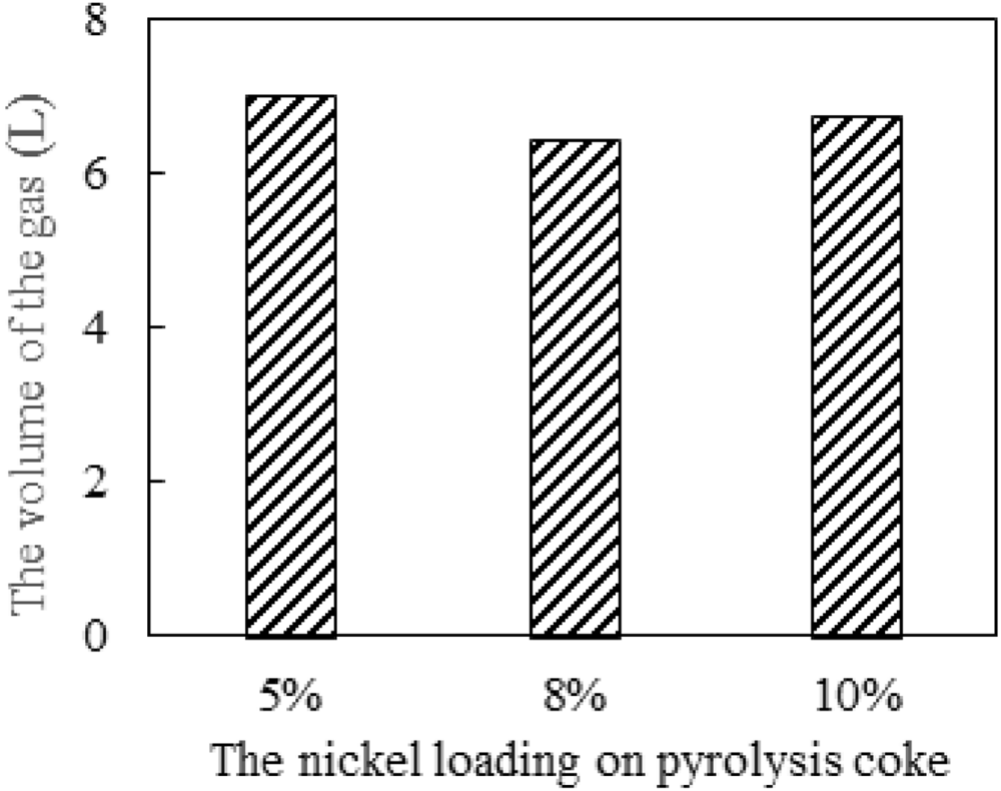
Effect of different nickel loading on gas yield by tar cracking.

**Figure 5:**
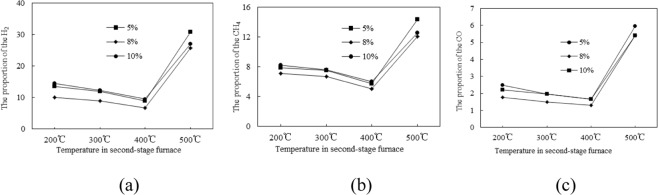
Effect of different nickel loading on the yield of H_2_, CH_4_ and CO.

The pyrolysis coke catalysts with different Ni loadings are placed in second-stage furnace to crack the tar, the gas yield by the tar cracking is measured, and the results are shown in Figs 4 and 5.

As can be seen from Figs 4 and 5, the gas yield by tar cracking is greatly increased after the nickel is supported on the pyrolysis coke catalyst. At a temperature of 500 °C, the proportions of combustible gas component (H_2_, CH_4_ and CO) in the gas are obviously increased. When the loading is 5%, the gas yield is the highest, which is 92% higher than when the pyrolysis catalyst is not used. At the same time, the proportion of combustible gas in the gas is also the highest. The reason may be that the nickel nitrate on the pyrolysis coke catalyst is sufficiently oxidized when the loading amount is 5%, and the generated nickel oxide can oxidize the gaseous hydrocarbon to generate hydrogen^17^. However, when the loading amounts are 8% and 10%, the nickel nitrate supported on the catalyst surface cannot be completely oxidized because the plasma treatment time is relatively short, so the catalytic effect is relatively poor, thus the optimum nickel loading can be determined to be 5%.

### Activity evaluation of bimetallic supported pyrolysis catalyst

**Figure 6:**
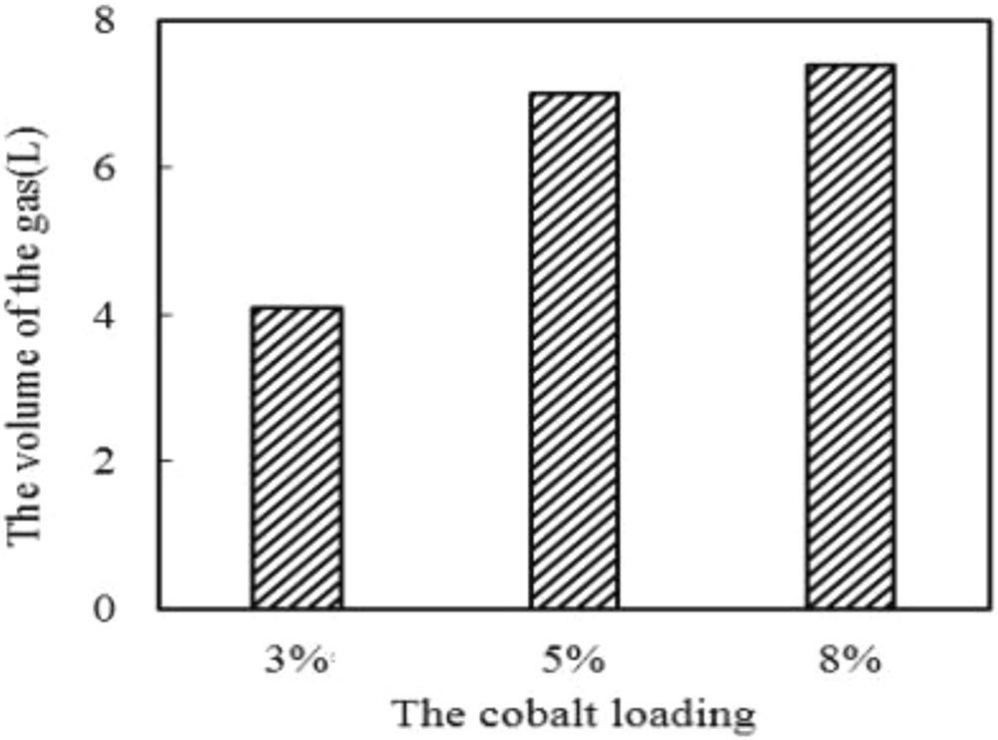
Effect of different cobalt loading on gas yield.

Based on the 5% nickel-loaded pyrolysis catalyst, the nickel-cobalt bimetallic supported pyrolysis catalyst was prepared by equal volume impregnation method. The cobalt loadings were 3%, 5% and 8%, respectively. Then catalytic experiments were carried out using the prepared bimetallic pyrolysis catalysts with different cobalt loadings. The experimental results are shown in Fig. 6.

**Figure 7:**
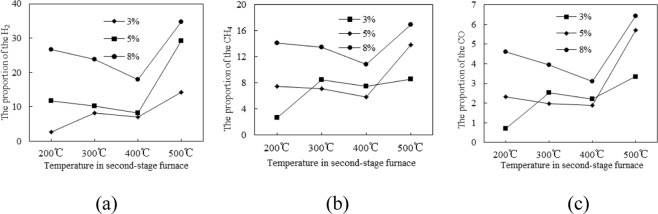
Effect of different cobalt loading on the yield of H_2_, CH_4_ and CO.

As can be seen from Figs 6 and 7, after the metal cobalt is supported on the pyrolysis coke catalyst with a nickel loading of 5%, the gas yield by tar cracking increases with the load amount increasing, and the gas yield by tar cracking is the highest when the cobalt loading is 8%, which is increased by 103% compared with none pyrolysis catalyst. At the same time, the proportion of flammable gas (H_2_, CH_4_ and CO) in the pyrolysis gas is also the highest. So the optimal cobalt loading is 8%. This is because Co forms a positive interaction with the catalytic component contained in the pyrolysis coke, and the H radical generated by the coal pyrolyze is effectively entered into the tar, thereby reducing the formation of heavy oil and increasing the yield of the light oil^16–19^.

## Characterization of the Pyrolysis Coke Catalyst

### BET analysis

#### BET analysis of the pyrolysis coke with different final temperature

Through BET analysis, we obtained specific surface area data of pyrolysis coke with different pyrolysis final temperature. The results are shown in Table 2.

**Table 2:** Specific surface area of pyrolysis coke with different final temperature.

Final temperature/°C	Specific surface area/(m^2^ · g^−1^)
450	6.85
550	15.47
650	59.54
750	88.71

As can be seen from Table 2, the specific surface area of the pyrolysis coke increase with the pyrolysis final temperature increasing. There are metal minerals in the pore structure of coal char, and the pores in the pyrolysis coke can prolong the retention time of the tar and promote its full binding with the active site of the pyrolysis catalyst to further catalyze the tar cracking^20–23^. The metal minerals in coal char are mainly alkali metals and alkaline earth metals. These metal ions are good tar cracking catalysts.

During the preparation of pyrolysis coke with higher pyrolysis temperature, the enriched minerals melt when it is coke, which is more conducive to the tar to cracking out more gas molecules. Therefore, when the raw coal pyrolysis product including tar and gas passes through the pyrolysis coke, the heavy components in the tar can be cracked to more light components, and the light components are decomposed into gases such as H_2_, CH_4_, and CO.

#### BET analysis of the pyrolysis coke catalyst with different load

The BET analysis of the original coke, the Ni supported catalyst and the Ni-Co supported catalyst was shown as Table 3.

**Table 3:** BET analysis of the pyrolysis coke catalyst with different load.

	The original coke	Ni supported catalyst	Mi-Co supported catalyst
the specific surface area (m^2^/g)	88.71	128.564	123.267
the total pore volume (cm^3^/g)	4.962	4.945	4.969
the pore size (nm)	4.62	3.97	4.27

As the Table 3 shows, the specific surface area of pyrolysis coke without supported metal is comparable to the specific surface area of single metal pyrolysis coke and the bimetal pyrolysis coke, so there is no direct correlation between the specific surface area of the catalyst and the catalytic activity. And because the pore size and the total pore volume of the three catalysts are basically the same. Therefore, it can be learned that the specific surface area, the pore size and the total pore volume are not the basic characterization of catalytic performance.

### XRD analysis

1. XRD analysis of the pyrolysis coke catalysts supported single metal
  In order to investigate the effect of metal loading on catalyst activity, XRD characterization of catalysts with different metal loadings is shown in Fig. 8.

**Figure 8:**
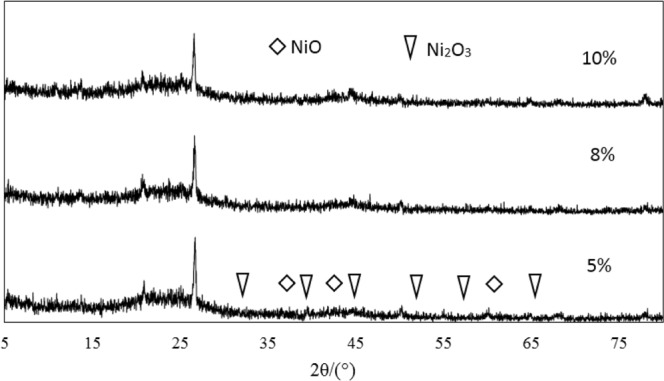
XRD analysis of pyrolysis coke catalysts with different nickel loading.
  As can be seen from Fig. 8, for the NiO/pyrolysis catalyst with a loading of 8% and 10%, the diffraction peak corresponding to the NiO phase can hardly be observed, this is because the metal oxide NiO on the NiO/pyrolysis catalyst was highly dispersed in the form of crystallites on the catalyst surface. In contrast, the NiO/pyrolysis catalyst with a loading of 5% exhibited weak diffraction peaks at 2θ = 37.325, 43.516, and 61.234, respectively, which are characteristic peaks of the metal oxide NiO. The shape of these three peaks is not sharp, which means that the dispersion of nickel oxide on the catalyst surface is relatively uniform. NiO plays a major role in the hydrogen production of tar cracked, it is a P-type semiconductor with considerable non-stoichiometric oxygen^24,25^. Since gaseous hydrocarbons are mainly produced in the pyrolysis stage at 400 °C to 500 °C, the formation of hydrogen is mainly due to the oxidative dehydrogenation of gaseous hydrocarbons, the mechanism of oxidative dehydrogenation of gaseous hydrocarbons on NiO can be inferred as: The gaseous hydrocarbon firstly reacts with the non-stoichiometric oxygen [O] in NiO to remove one -H to form a hydrocarbyl radical, and then further de-H to form a lower order hydrocarbon^26^. The reaction equation for this process is:
  12 $${{\rm{C}}}_{{\rm{n}}}{{\rm{H}}}_{2{\rm{n}}+2}+[{\rm{O}}]\to {{\rm{C}}}_{{\rm{n}}}{{\rm{H}}}_{2{\rm{n}}+1}+{{\rm{OH}}}^{-}$$
  13 $${{\rm{C}}}_{{\rm{n}}}{{\rm{H}}}_{2{\rm{n}}+2}+[{\rm{O}}]\to {{\rm{C}}}_{{\rm{n}}}{{\rm{H}}}_{2{\rm{n}}}+{{\rm{OH}}}^{-}$$
  14 $${{\rm{OH}}}^{-}+{{\rm{OH}}}^{-}\to {{\rm{H}}}_{2}+2[{\rm{O}}]$$
2. XRD analysis of Bimetallic supported pyrolysis coke catalysts

**Figure 9:**
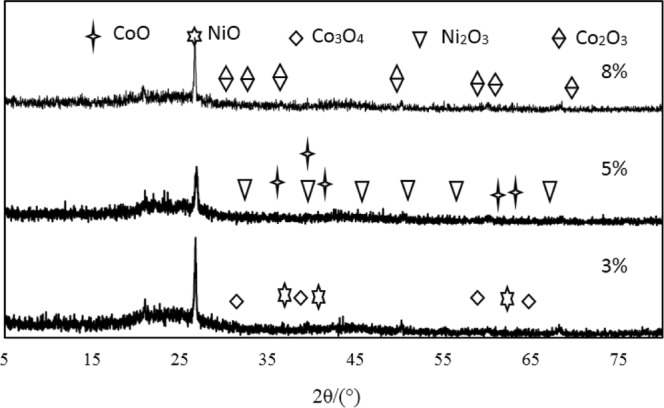
XRD analysis of the bimetallic catalysts with different cobalt loading.

The nickel-cobalt bimetallic supported pyrolysis catalyst was prepared based on the 5% nickel-loaded pyrolysis catalyst with the cobalt loadings of 3%, 5% and 8%, respectively. The XRD analysis of the nickel-cobalt bimetallic supported pyrolysis catalyst was shown in Fig. 9.

As can be seen from Fig. 9, the reason why the catalytic activity of the bimetallic supported catalyst is superior to that of the single metal supported catalyst is that Co_3_O_4_ is a P-type semiconductor and is electrically conductive by holes. The holes of Co_3_O_4_ are increased, the conductivity of the P-type semiconductor is increased after loading Co_3_O_4_ on NiO. When an alkane is adsorbed on a catalyst to become a positive ion, electrons are given to a P-type semiconductor, which reduces the number of holes in the conduction. The reduction in the number of holes is not conducive to receiving electrons from propane^27^.

If nickel is added to the acceptor substance, the number of holes will increase. If nickel is added to the acceptor substance, the number of holes and the conductivity will both increase, which will facilitate the adsorption on the surface, and correspondingly reduce the activation energy of hydrogen production during the secondary cracking of the alkane.

### XPS analysis

1. XPS analysis of single metal supported pyrolysis coke catalysts
  In order to further determine the energy of the metal oxide in the catalyst, it was characterized by XPS, and the XPS spectrum of the single metal supported catalyst is shown in Fig. 10.

**Figure 10:**
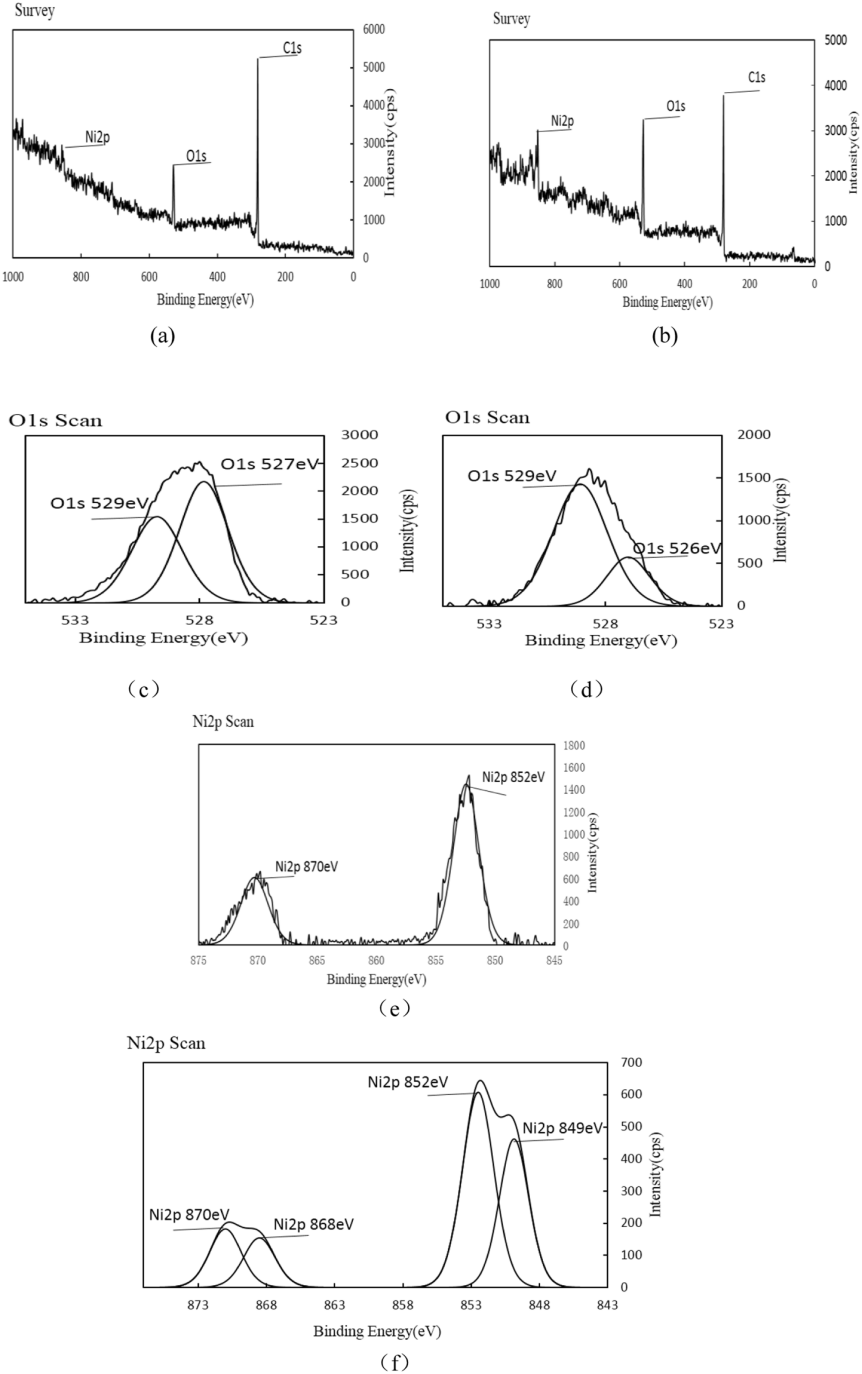
XPS spectra before and after reaction of pyrolysis coke catalyst with nickel loading of 5%.
  As can be seen from the Fig. 10(c), it can be seen from the peak of oxygen, there are two forms of oxygen in the catalyst, namely 527 eV lattice oxygen and 529 eV chemisorbed oxygen, and the lattice oxygen intensity is significantly higher than that of the chemical adsorption of oxygen. The intensity of the lattice oxygen in the graph (d) is lower than that of the chemisorbed oxygen, which indicates that the lattice oxygen participates in the reaction during the reaction, and the nickel on the surface of the catalyst is oxidized to the oxide of nickel. It can be seen from Fig. 10(e,f) that there are two peaks of metallic nickel on the catalyst surface, indicating the presence of two different valence nickel oxides in the catalyst.
  Among them, 852 eV refers to Ni^3+^, and 870 eV refers to Ni^2+^. Comparing Fig. 10(e) with (f), it was found that the strength of Ni^2+^ and Ni^3+^ decreased after the reaction, indicating that NiO played a major role in the reaction. We have learned that the catalyst contains more trivalent nickel and a small amount of divalent nickel according to the XRD spectrum above, and XPS result can well verify this^28,29^. This indicates that it is easier to generate a highly active metal oxide on the surface of the bimetallic catalyst during the plasma calcination, which is advantageous for promoting the cracking of the hydrocarbon by the catalyst.
2. XPS analysis of bimetallic supported pyrolysis coke catalysts

**Figure 11:**
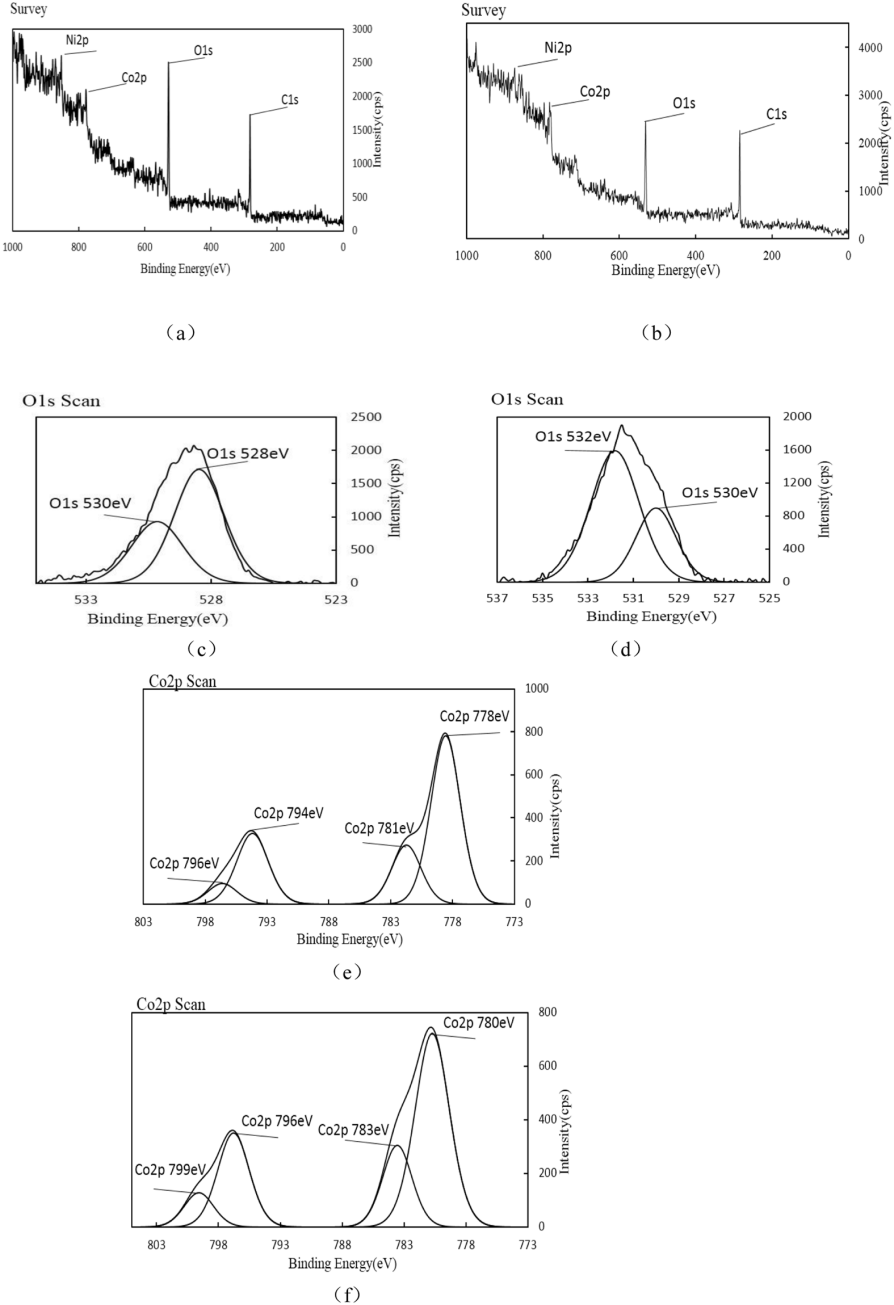
XPS spectrum before and after the reaction of the bimetallic supported catalyst.

To characterize the oxidation morphology of the metal in the bimetallic (Ni-Co) supported catalyst, it was characterized by XPS. The XPS spectrum before and after the reaction of the bimetallic supported catalyst is shown in Fig. 11.

It can be seen from Fig. 11(c,d), after the reaction of the bimetallic supported catalyst, the strength of the lattice oxygen is lower than the intensity of the chemically adsorbed oxygen. It can be seen from Fig. 11(e,f) that there are two peaks of metallic cobalt on the catalyst surface, that is, there are two oxides having different valence states, and loading the metallic cobalt on the basis of the nickel-based catalyst can improve the state of oxides and oxygen of metallic nickel in the catalyst.

At the same time, the amount of adsorbed oxygen in the catalyst is changed from lower than lattice oxygen to higher than lattice oxygen. The activity of the catalyst is obviously improved after the addition of metallic cobalt, because the addition of cobalt significantly increases the amount of adsorbed oxygen in the catalyst, and the chemically adsorbed oxygen can increase the number of holes in the P-type semiconductor and reduce the reaction energy in the catalytic reaction process and it has a good promoting effect on low temperature reactivity. Combined with the influence of the bimetallic supported catalyst on the pyrolysis products, it can be seen that in the catalytic process, the adsorbed oxygen in the catalyst does not directly participate in the reaction, but promotes the pyrolysis reaction by changing the reaction energy in the reaction process^30^. Moreover, chemically adsorbed oxygen may be more favorable for the low temperature catalytic activity of the catalyst.

### SEM analysis

1. SEM analysis of single metal supported pyrolysis coke catalysts
  The morphology of the single metal supported catalyst before reaction is shown in Fig. 12(a) and the morphology of the single metal supported catalyst after reaction is shown in Fig. 12(b).

**Figure 12:**
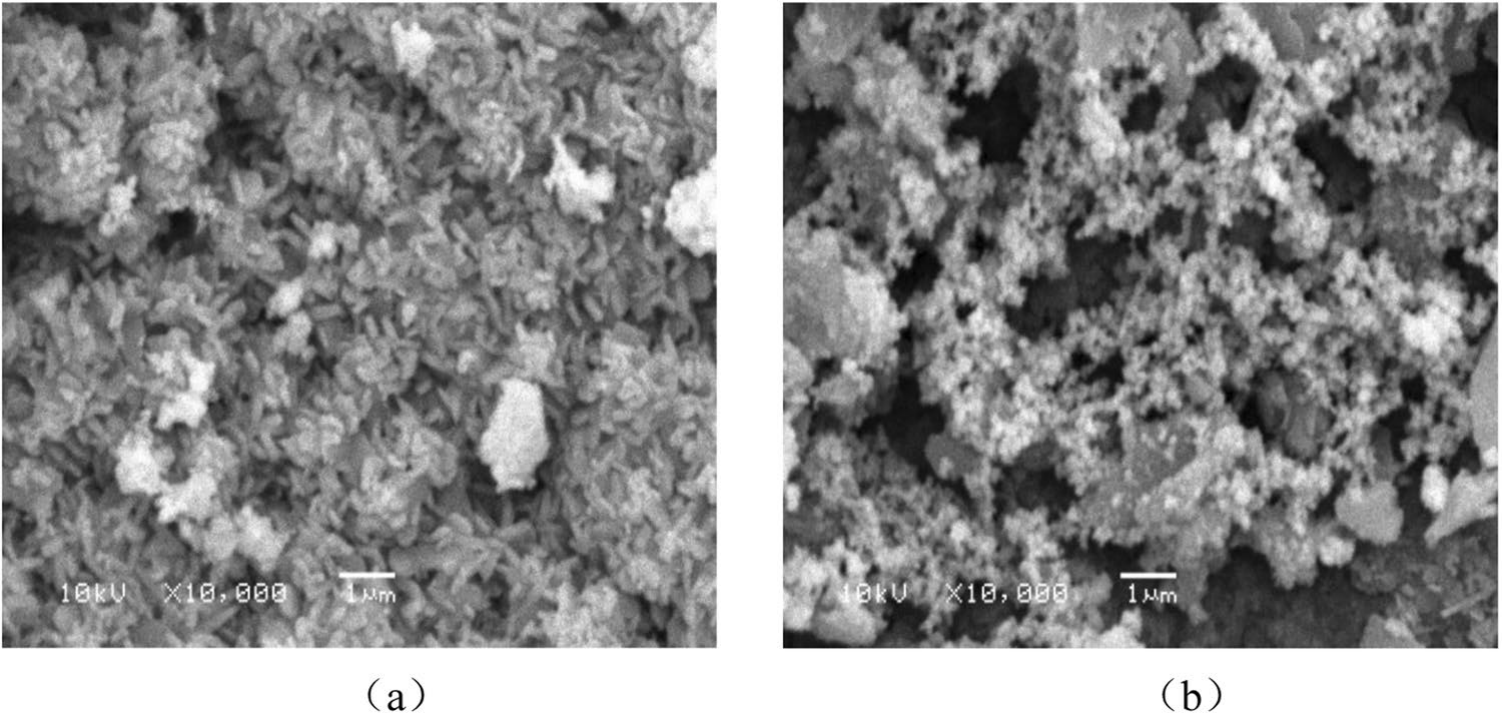
SEM spectrum before and after reaction of the single metal catalyst.
  It can be seen from the Fig. 12(a) that the surface-distribution of the nickel-supported pyrolysis coke after plasma modification-baking is uniform, and it can be inferred that these crystals are nickel oxides combine with the XRD spectrum of the single-metal catalyst. In Fig. 12(b), the number of crystals is significantly reduced, and the crystals are scorched together and unevenly distributed. This may be because the tar adheres to the surface of the catalyst and reacts, resulting in the number of pores on the surface of the catalyst is also relatively reduced^31–33^.
2. SEM analysis of bimetallic supported pyrolysis coke catalysts

**Figure 13:**
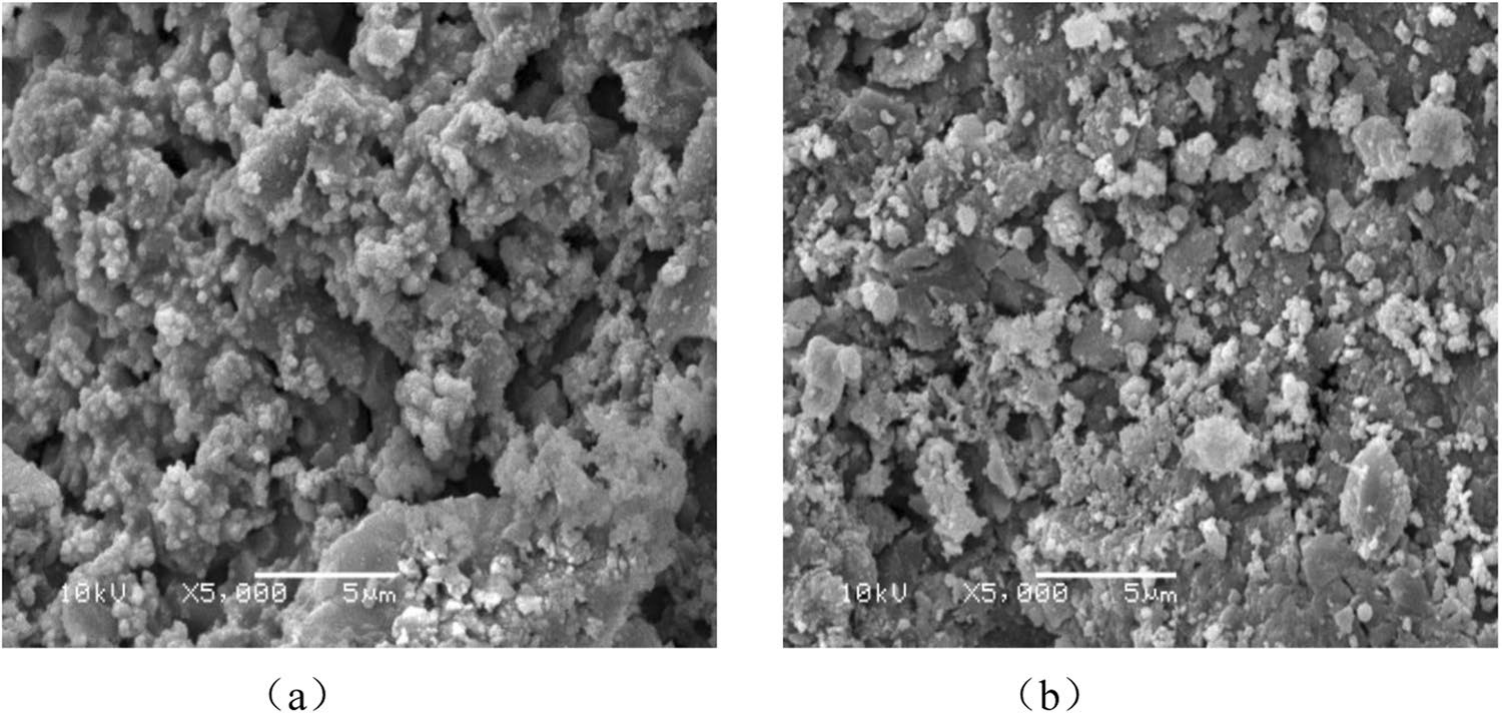
SEM spectrum before and after reaction of the bimetallic catalyst.

The morphology of the bimetallic supported catalyst before reaction is shown in Fig. 13(a,b) correspond to the surface topography before and after the catalyst reaction, respectively.

It can be seen from Fig. 13(a) that after the plasma modification-baking of the bimetallic catalyst, a large amount of crystals appear on the surface of the catalyst and the amount of pores is increased compared to the single metal supported catalyst. New particles appear on the surface of the catalyst after the reaction, and it is inferred from the XRD spectrum that these crystals may contain nickel oxides and cobalt oxides^34,35^. The number of crystals in Fig. 13(b) is significantly reduced, indicating that some of the nickel oxide and cobalt oxide are involved in the tar cracking process^36^.

## Conclusion

In this study, the single metal supported pyrolysis catalyst was prepared by using pyrolysis coke as a carrier and loading 5%, 8%, and 10% nickel on the surface by an equal volume impregnation method. By investigating the effect of different nickel loadings on the pyrolysis gases yield, the best-performing single-metal supported catalysts were screened, and then 3%, 5%, 8% of the cobalt was loaded on the surface. The best Ni-Co bimetallic supported catalysts were screened by experiment. The conclusions are as below:

1. The optimal loading of metallic nickel is 5%. Compared with the blank group, the coal pyrolysis gas production is increased by 92%, and the proportion of flammable gas in the gas product is also increased;
2. When the metal nickel loading is 5% and the metal cobalt loading is 8%, the obtained bimetallic supported pyrolysis catalyst has the best activity. Metal nickel and cobalt in the bimetallic catalyst produced a synergistic effect and formed a P-type semiconductor, and the yield of pyrolysis gas increased by 103% over the blank group.
